# Lignocellulosic agriculture wastes as biomass feedstocks for second-generation bioethanol production: concepts and recent developments

**DOI:** 10.1007/s13205-014-0246-5

**Published:** 2014-08-21

**Authors:** Jitendra Kumar Saini, Reetu Saini, Lakshmi Tewari

**Affiliations:** 1 Department of Microbiology, College of Basic Sciences and Humanities, GB Pant University of Agriculture and Technology, Pantnagar, Udham Singh Nagar, 263145 India; 2 Department of Microbiology, M.S. Garg P.G. College, Laksar, Haridwar, 247663 India; 3 Present Address: DBT-IOC Centre for Advanced Bio-Energy Research, Research and Development Centre, Indian Oil Corporation Ltd., Sector-13, Faridabad, 121007 Haryana India

**Keywords:** Lignocellulose, Bioethanol, Cellulase, Agricultural wastes, Residues

## Abstract

Production of liquid biofuels, such as bioethanol, has been advocated as a sustainable option to tackle the problems associated with rising crude oil prices, global warming and diminishing petroleum reserves. Second-generation bioethanol is produced from lignocellulosic feedstock by its saccharification, followed by microbial fermentation and product recovery. Agricultural residues generated as wastes during or after processing of agricultural crops are one of such renewable and lignocellulose-rich biomass resources available in huge amounts for bioethanol production. These agricultural residues are converted to bioethanol in several steps which are described here. This review enlightens various steps involved in production of the second-generation bioethanol. Mechanisms and recent advances in pretreatment, cellulases production and second-generation ethanol production processes are described here.

## Introduction

One of the greatest challenges of twenty-first century is to meet the growing demand of energy for transportation, heating and industrial processes, and to provide raw materials for chemical industries in sustainable ways. Biofuels have emerged as an ideal option to meet these requirements in a sustainable manner. Several primary drivers underlie the increasing interests in biofuels, such as increasing uncertainty of petroleum supplies due to rising demand, decline in known reserves, and concerns over global warming and green house gas emissions associated with fossil fuels usage and this has led to various government programs promoting biofuels. Moreover, biofuels are unique among available alternative energy sources in their general compatibility with existing liquid transport fuel. The global production and use of biofuels have increased dramatically in recent years, from 18.2 billion liters in 2000 to 60.6 billion liters in 2007, with about 85 % of this being bioethanol. Bioethanol is the most common and one of the practically important liquid biofuel and can be produced from a variety of cheap substrates. According to an estimate, it can reduce greenhouse gas emissions by approximately 30–85 % compared to gasoline, depending on the feedstock used (Fulton et al. 2004). The USA and Brazil are currently the primary producers of fuel ethanol, producing 49.6 and 38.3 % of 2007 global production, respectively (Coyle 2007). Worldwide increasing interest in the production of bioethanol is exemplified by production of 85 billion liters of bioethanol in 2011 (Singh and Bishnoi 2012; Avci et al. 2013).

The present review is a concise overview of the basic concepts and some recent advances in ethanol production with special emphasis on lignocellulosic agricultural residues/wastes and their sources, pretreatment methods, enzymatic hydrolysis and fermentation to generate bioethanol in ecologically sustainable and cost-effective manner. Some challenges still existing in economic production of second-generation bioethanol and their potential solutions are discussed in brief towards the end.

## Bioethanol: an eco-friendly biofuel

Bioethanol is made biologically by fermentation of sugars derived from a variety of sources. The use of ethanol as a motor fuel began with its use in the internal combustion engine invented by Nikolas Otto in 1897 (Ahindra 2008). Alcohols have been used as fuels since the inception of the automobile. The term alcohol often has been used to denote either ethanol or methanol as a fuel. With the oil crises of 1970s, ethanol became established as an alternative fuel. Many countries started programs to study and develop fuels in an economic way from available raw materials. Countries including Brazil and the USA have long promoted domestic bioethanol production. “First generation bioethanol” is made from sugar feedstock such as cane juice (in Brazil) and molasses (in India) or from starch-rich materials such as corn (in US). Though bioethanol production from ‘first generation technologies’ is estimated to increase to more than 100 billion liters by 2022 (Goldemberg and Guardabasi 2009), these raw materials compete with food, are insufficient to meet the increasing demand for fuels, have negative impact on biodiversity and may even lead to deforestation to gain more farmland (Hahn-Hägerdal et al. 2006). The cumulative impact of these concerns have increased the interests in developing “second generation ethanol” from non-food lignocellulosic materials such as agricultural residues, wood, paper and municipal solid waste, and dedicated energy crops (viz. miscanthus, switchgrass, sweet sorghum, etc.), which constitute the most abundant renewable organic component in the biosphere (Claassen et al. 1999).

Bioethanol is widely recognized as a unique transportation fuel with powerful economic, environmental and strategic attributes. As bioethanol can be produced from biomass of crop plants, it offers opportunities to improve the income levels of smallholder farmers. At a community level, farmers can cultivate energy crops that fetch an income while also meeting their food needs. Ethanol derived from biomass is the only liquid transportation fuel that does not contribute to the green house gas effect. Ethanol represents closed carbon dioxide cycle because after burning of ethanol, the released carbon dioxide is recycled back into plant material as plants use it to synthesize cellulose during photosynthesis. Ethanol production process only uses energy from renewable energy sources; no net carbon dioxide is added to the atmosphere, making ethanol an environmentally beneficial energy source. Ethanol contains 35 % oxygen that helps complete combustion of fuel and thus reduces particulate emission that poses health hazard to living beings. The toxicity of the exhaust emissions from ethanol is lower than that of petroleum sources (Wyman and Hinman 1990). Thus, the use of even 10 % ethanol blends reduces GHG emissions by 12–19 % compared with conventional fossil fuels. Burning E 85 (85 % ethanol) reduces the nitrogen oxide, particulate and sulfate emissions by 10, 20 and 80 %, respectively, compared to conventional gasoline.

Bioethanol market is expected to reach 10 × 10^10^ l in 2015 (Licht 2008). The largest bioethanol producers in the world are the US, Brazil, and China. In 2009, US produced 39.5 × 10^9^ l of ethanol using corn as a feedstock while the second largest producer, Brazil, created about 30 × 10^9^ l of ethanol using sugarcane. China is nowadays investing heavily in ethanol production and is one of its largest producers (Ivanova et al. 2011). In India, the interest in biofuels is growing so as to substitute oil for achieving energy security and promote agricultural growth. Indian government has planned to achieve a target of 20 % blending of fossil fuels with biodiesel and bioethanol by 2017. In addition, a national policy for biofuel has been framed including promotion of biofuel production, particularly on wastelands (Ravindranath et al. 2011).

### Feedstocks for bioethanol: agricultural residues

The varied raw materials used in the manufacture of bioethanol are conveniently classified into three main types: sugars, starches, and cellulose materials. Sugars (such as cane or sweet sorghum juice, molasses) can be used directly for ethanol production via fermentation. Starches (from corn, cassava, potatoes, and root crops) must first be hydrolyzed to fermentable sugars by the action of enzymes from malt or molds. Cellulose (from wood, agricultural residues, waste sulfite liquor from pulp, and paper mills) must likewise be converted into sugars, generally by the action of acids or cellulolytic enzymes (Franks et al. 2006).

There are various forms of biomass resources in the world, which can be grouped into four categories, viz. wood product industry wastes, municipal solid waste, agriculture residues and dedicated energy crops. These biomass resources seem to be the largest and most promising future resources for biofuels production. This is because of the ability to obtain numerous harvests from a single planting, which significantly reduces average annual costs for establishing and managing energy crops, particularly in comparison to conventional crops (Franks et al. 2006). The global production of plant biomass, of which over 90 % is lignocellulose, amounts to about 200 × 10^9^ tons/year, where about 8–20 × 10^9^ tons of the primary biomass remains potentially accessible (Kuhad and Singh 1993). Lignocellulosic material represents a promising option as feedstock for ethanol production considering their output/input energy ratio, availability, low cost and higher ethanol yields. For second-generation biofuel production, utilization of renewable biomass resources has received major focus in the world. Renewable ‘plant biomass’ refers particularly to cheap and abundant non-food lignocellulose-rich materials available from the plants. Biomass to bioethanol process could help in mitigation of global climate change by reducing emissions (mainly CO_2_) as well as decreasing dependence upon fossil fuels. Thus, deployment of biomass resources has been projected to play an important role in sustainable development. The second-generation biofuels include hydrogen, natural gas, bio-oils, producer gas, biogas, alcohols and biodiesel. In countries like India, agricultural production of various crops like cotton, mustard, chilli, sugarcane, sorghum, sweet sorghum, pulses, oilseeds, etc. results in generation of huge amounts of wastes that do not find any alternative use and are either left in the fields or are burned. Hence, these could be used as good alternative resources to generate biofuels such as bioethanol, in an environmentally friendly manner. Use of agricultural residues helps in reduction of deforestation by decreasing our reliance on forest woody biomass. Moreover, crop residues have short harvest period that renders them more consistently available to bioethanol production (Knauf and Moniruzzaman 2004; Kim and Dale 2004; Limayema and Ricke 2012).

Maize, wheat, rice, and sugarcane are the four agricultural crops with maximum production as well as area under cultivation. These four crops are responsible for generating majority of lignocellulosic biomass in agriculture sector and rest of the agrowastes constitute only a minor proportion of the total agrowaste production in the world. Corn stover is the left over residue after harvesting corn kernel and comprises stalks, leaves, cobs, and husks. Its annual production is approximately 1 kg/kg corn grain or 4 tons/acre (Kim and Dale 2004; Heaton et al. 2008; Cheng and Timilsina 2011). Straw is generated during wheat grain harvest at a rate of 1–3 tons/acre annually under rigorous farming conditions. Rice straw is the leftover of rice production and includes stems, leaf blades, leaf sheaths, and the remains of the panicle after threshing. It is one of the most abundant lignocellulosic waste materials in the world. Out of the annual global production of 731 million tons of rice straw Asia alone produces 667.6 million tons. Bagasse is produced in huge amounts during sugarcane processing. It is also a cheap renewable agricultural resource for ethanol production (Bhatia and Paliwal 2011). Most of the agricultural residues have similar contents of cellulose, hemicelluloses, and lignin, but rice straw has more silica content while wheat straw contains significant amount of pectin and proteins (Sarkar et al. 2012).

Lignocellulosic agricultural wastes have cellulose as a major component, but their chemical composition varies considerably (Table 1). Global production of major agrowastes and their bioethanol production potential are shown in Table 2. Maximum rice straw and wheat straw are generated in Asia and corn straw and sugarcane bagasse are mainly produced in America. According to an estimate, lignocellulosic biomass can be used to generate approximately 442 billion liters of bioethanol per year and if total crop residues and wasted crops are also considered, this figure can rise to 491 billion liters, about 16 times higher production than the actual global production (Kim and Dale 2004; Sarkar et al. 2012). In US alone, a total of 1,368 MT biomass are available for bioethanol production, out of which agrowastes with 428 MT constitute major proportion, followed by forestry wastes, energy crops, grains and corn, municipal and industrial wastes and other wastes contributing 370, 377, 87, 58 and 48 MT, respectively (Perlack et al. 2005; USDOE Biomass Program 2009; RFA 2010).

**Table 1:** Composition of various agricultural and other lignocellulosic residues

Material	Cellulose^a^	Hemicellulose	Lignin	Ash	Extractives
Algae (green)	20–40	20–50	–	–	–
Cotton, flax, etc.	80–95	5–20	–	–	–
Grasses	25–40	25–50	10–30	–	–
Hardwoods	45 ± 2	30 ± 5	20 ± 4	0.6 ± 0.2	5 ± 3
Hardwood barks	22–40	20–38	30–55	0.8 ± 0.2	6 ± 2
Softwoods	42 ± 2	27 ± 2	28 ± 3	0.5 ± 0.1	3 ± 2
Softwood barks	18–38	15–33	30–60	0.8 ± 0.2	–
Cornstalk	39–47	26–31	3–5	12–16	–
Wheat straw	37–41	27–32	13–15	11–14	–
Newspaper	40–55	25–40	18–30	–	–
Chemical pulp	60–80	20–30	2–10	–	–
Sorghum stalks	27	25	11	–	–
Corn stover	38–40	28	7–21	3.6–7.0	–
Coir	36–43	0.15–0.25	41–45	2.7–10.2	–
Bagasse	32–48	19–24	23–32	1.5–5	–
Rice straw	28–36	23–28	12–14	14–20	–
Wheat straw	33–38	26–32	17–19	6–8	–
Barley straw	31–45	27–38	14–19	2–7	–
Sorghum straw	32	24	13	12	–
Sweet sorghum Bagasse	34–45	18–28	14–22	–	–

*Ref* Kuhad et al. (1997), Reddy and Yang (2005), Li et al. (2010)

^a^Composition represented in %wt on dry matter basis

**Table 2:** Worldwide availability of major agricultural wastes and their bioethanol production potential

Agricultural wastes	Availability^a^ (million tons)	Estimated bioethanol potential^a^ (Gl)
Wheat straw	354.34	104
Rice straw	731.3	205
Corn straw	128.02	58.6
Sugarcane bagasse	180.73	51.3

^a^Calculated from Sarkar et al. (2012)

### Structural organization of lignocellulosic feedstocks

Agricultural residues such as wheat straw, rice straw, bagasse, cotton stalk and wheat bran are rich in lignocellulose and primarily contain cellulose, lignin, hemicellulose, and extractives. Cellulose forms a skeleton that is surrounded by hemicellulose and lignin functioning as matrix and encrusting materials, respectively (Ingram and Doran 1995). Table 1 presents the biochemical composition of major lignocellulosic feedstocks that are being used worldwide for bioethanol production.

#### Cell wall polysaccharides

Classically, cell wall polysaccharides have been grouped into three fractions: cellulose, hemicellulose and pectic polysaccharides, proteins and other miscellaneous compounds (Chesson and Forsberg 1988) as discussed below.

##### Cellulose

Cellulose, the major structural component in the plant cell wall, is a linear homo-polysaccharide consisting of anhydrous glucose units (500–15,000) that are linked by β-1,4-glycosidic bonds, with cellobiose as the smallest repetitive unit. The β-1,4 orientation of the glucosidic bonds results in the potential formation of intramolecular and intermolecular hydrogen bonds, which make native cellulose highly crystalline, insoluble, and resistant to enzyme attack. The highly crystalline regions of cellulose in the plant cell wall are separated by less ordered amorphous regions (Chesson and Forsberg 1988).

##### Hemicellulose

Hemicellulose is a short, highly branched polymer of pentoses (e.g. d-xylose and l-arabinose) and hexoses (e.g. d-manose, d-galactose, and d-glucose) with 50–200 units. Its acetate groups were randomly attached with ester linkages to the hydroxyl groups of the sugar rings. The role of hemicellulose is to provide a linkage between lignin and cellulose (Holtzapple 1993).

##### Pectic compounds and proteins

Pectic polysaccharides make up approximately 35 % of the primary cell walls, the main components being galactosyluronic residues. Its other major components are rhamnose, arabinose, and galactose. Pectic substances are hydrophillic and therefore have certain adhesive properties. Proteins are a minor component of the plant cell wall which may be covalently cross-linked with lignin and polysaccharides (Cassab and Varner 1988).

##### Phenolic compounds

Three types of phenolic compounds viz. lignin, tannins and phenolic acids are found in plant cell walls. Lignin is a heterogeneous, amorphous, and cross-linked aromatic polymer where the main aromatic components are trans-coniferyl, trans-sinapyl and trans-*p*-coumaryl alcohols. Lignin is covalently bound to side groups on different hemicelluloses, forming a complex matrix that surrounds the cellulose micro-fibrils. In plant cell wall it varies from 2 to 40 %. The existence of strong carbon–carbon (C–C) and ether (C–O–C) linkages in the lignin gives the plant cell wall strength and protection from attack by cellulolytic microorganisms (Mooney et al. 1998). Tannins are high molecular weight (500–3,000) polyphenolic compounds, composed of either hydroxyflavans, leucoanthocyanidin (flavan-3,4-diol) and catechin (flavan-3-ol) or glucose. Phenolic acids are structural components of the lignin core in plant cell wall. The presence of carboxyl and phenolic groups in phenolic acids enable such compounds to link to lignin and carbohydrates by ether or ester bonds.

## Bioconversion of lignocellulosic biomass to bioethanol

Biomass to ethanol bioconversion process consists of several steps, including pretreatment of biomass, enzymatic hydrolysis, fermentation and product recovery. Proper combination of each step is important for achieving higher bioethanol yield in a cost-effective and sustainable manner.

### Processing and pretreatments

The main processing challenge in the ethanol production from lignocellulosic biomass is the feedstock pretreatment. During pretreatment, the matrix of cellulose and lignin bound by hemicellulose should be broken to reduce the crystallinity of cellulose and increase the fraction of amorphous cellulose, the most suitable form for enzymatic attack. The yield of cellulose hydrolysis after pretreatment often exceeds 90 % of theoretical as compared to 20 % when pretreatment is not carried out (Lynd 1996). For pretreatment of lignocellulosics, several physical, physico-chemical and biological processes have been developed that improve lignocellulose digestibility in very different ways (Aden et al. 2002; Sun and Cheng 2002; Wyman et al. 2005a, b). These processes are summarized in Table 3.

**Table 3:** Pretreatment methods of lignocellulosic biomass for fuel ethanol production

Methods	Procedure/agents	Remarks	Examples of pretreated materials	References
I*. Physical methods*				
Mechanical size reduction	Chipping, grinding, milling	Milling: vibratory ball mill Wiley mill (final size: 0.2–2 mm), knife or hammer mill (final size: 3–6 mm)	Hardwood, straw, corn stover, timothy, alfalfa, cane and sweet sorghum bagasse	Sun and Cheng (2002)
Pyrolysis	*T* > 300 °C, then cooling and condensing	Formation of volatile products and char Residues; produce 80–85 % reducing sugars (>50 % glucose); can be carried out under vacuum	Wood, Waste cotton, corn stover	Khiyami et al. (2005)
II*. Physico*–*chemical methods*				
Steam explosion	Saturated steam at 160–290 °C, *p* = 0.69–4.85 MPa for several sec or min, then decompression until atm. Pressure	It can handle high solid loads; size reduction with lower energy input compared to comminution, 80–100 % hemicellulose hydrolysis, destruction of a portion of xylan fraction, 45–65 % xylose recovery; Inhibitors formation; addition of H_2_SO_4_, SO_2_, or CO_2_ improves efficiency of further enzymatic hydrolysis; cellulose depolymerization	Poplar, aspen, eucalyptus softwood (Douglas fir) bagasse, corn stalk, wheat straw, rice straw, barley straw, sweet sorghum bagasse, *Brassica carinata* residue, olive stones, Timothy grass, alfalfa, reed canary grass	Ballesteros et al. (2001, 2002, 2004), Hamelinck et al. (2005), Lynd et al. (2002), Soderstrom et al. (2005), Sun and Cheng (2002)
Liquid hot water (LHW)	Pressurized hot water, *p* > 5 MPa, *T* = 170–230 °C, 1–46 min; solids load <20 %	Lignin is not solubilized, but redistributed; 80–100 % hemicellulose hydrolysis, 88–98 %xylose recovery; low or no formation of inhibitors; cellulose conversion >90 %; partial solubilization of lignin (20–50 %)	Bagasse, corn stover, olive pulp, Alfalfa fiber	Ballesteros et al. (2002), Koegel et al. (1999), Lynd et al. (2002)
Ammonia fiber explosion (AFEX)	1–2 kg ammonia/kg dry biomass, 90 °C, 30 min, *p* = 1.12–1.36 MPa	Ammonia recovery is required 0–60 % hemicellulose hydrolysis; no inhibitor formation; further cellulose conversion can be >90 %, for high-lignin biomass (<50 %); 10–20 % lignin solubilization	Aspen wood chips bagasse, wheat straw, barley straw, rice hulls, corn stover switchgrass, coastal bermudagrass, alfalfa newsprint	Lynd et al. (2002), Sun and Cheng (2002)
CO_2_ explosion	4 kg CO_2_/kg fiber, *p* = 5.62 MPa	No inhibitors formation Further cellulose conversion can be >75 %	MSW Bagasse Alfalfa recycled paper	Sun and Cheng (2002)
Ozonolysis	Ozone, room temperature and pressure	No inhibitors formation further cellulose conversion can be >57 % lignin degradation	Poplar, sawdust, pine, bagasse, wheat straw, cotton straw, green hay, peanut	Sun and Cheng (2002)
Dilute-acid hydrolysis	0.75–5 % H_2_SO_4_, HCl, or HNO3, *p* = 1 MPa; continuous process for low solids loads (5–10 wt% substrate/mixture); *T* = 160–200 °C; batch process for high solids loads (10–40 % substrate/mixture); *T* = 120–160 °C	pH neutralization is required that generates gypsum as a residue; 80–100 % hemicellulose hydrolysis, 75–90 % xylose recovery; high temperature favors further cellulose hydrolysis lignin is not solubilized, but it is redistributed	Poplar wood bagasse, corn stover, wheat straw, rye straw, rice hulls, switchgrass, Bermudagrass	Hamelinck et al. (2005), Lynd et al. (2002), Sun and Cheng (2002), Wooley et al. (1999)
Concentrated-acid hydrolysis	10–30 % H_2_SO_4_, 170–190 °C, 1:1,6 solid–liquid ratio 21–60 % peracetic acid, silo-type system	Acid recovery is required; residence time greater compared to dilute-acid hydrolysis; peracetic acid provokes lignin oxidation	Poplar sawdust, bagasse	Cuzens and Miller (1997)
Alkaline hydrolysis	Dilute NaOH, 24 h, 60 °C; Ca(OH)_2_, 4 h, 120 °C; it can be complemented by adding H_2_O_2_ (0.5–2.15 vol.%) at lower temperature (35 °C)	Reactor costs are lower compared to acid pretreatment >50 % hemicellulose hydrolysis, 60–75 % xylose; recovery low inhibitors formation; cellulose swelling; further cellulose conversion can be >65 %; 24–55 % lignin removal for hardwood, lower for softwood	Hardwood, bagasse, corn stover, straws with low lignin content (10–18 %), cane leaves	Hamelinck et al. (2005), Kaar and Holtzapple (2000), Lynd et al. (2002), Saha and Cotta (2008), Sun and Cheng (2002)
Organosolv process	Organic solvents (methanol, ethanol, acetone, ethylene glycol, triethylene glycol) or their mixture with 1 % of H_2_SO_4_ or HCl; 185–98 °C, 30–60 min, pH = 2.0–3.4	Solvent recovery required; almost total hydrolysis of hemicellulose; high yield of xylose almost total lignin solubilization and breakdown of internal lignin and hemicellulose bonds	Poplar wood mixed softwood (spruce, pine, Douglas fir)	Lynd et al. (2002), Pan et al. (2005), Sun and Cheng (2002)
III. *Biological methods*				
Fungal pretreatment	Brown-, white- and soft-rot fungi; Cellulase and hemicellulase production by solid-state fermentation of biomass	Fungi produces cellulases, hemicellulases, and lignin-degrading enzymes: ligninases, lignin. peroxidases, polyphenoloxidases, laccase and quinone-reducing enzymes; very slow process	Corn stover, wheat straw	Sun and Cheng (2002)
Bioorganosolv pretreatment	*Ceriporiopsis subvermispora* for 2–8 weeks followed by ethanolysis at 140–200 °C for 2 h	Fungi decompose the lignin network ethanol action allows hemicellulose hydrolysis biological pretreatment can save 15 % of the electricity needed for ethanolysis ethanol can be reused; environmentally friendly process	Beech wood	Itoh et al. (2003)

#### Physical pretreatment methods

Lignocellulosic biomass can be pulverized by chipping, grinding, shearing, or milling, which reduces the particle size and increases surface area, facilitating the access of cellulases to the biomass surface and increasing the conversion of cellulose. Primary size reduction employs hammer mills or Wiley mills to produce particles that can pass through 3- to 5-mm diameter sieve. Other useful physical treatment methods include pyrolysis, irradiation with gamma radiation, microwave, infrared, or sonication (Brown 2003; Mosier et al. 2005).

#### Physico-chemical methods

Physico-chemical methods are considerably more effective than physical methods of pretreatment. Different chemical agents employed during these processes are ozone, acids, alkali, peroxide and organic solvents. Several physico-chemical methods (Table 2) are employed for pretreament of biomass before its saccharification, such as ammonia fiber explosion (AFEX) (Sun and Cheng 2002; Mosier et al. 2005), autohydrolysis (steam explosion) (Grous et al. 1986; Ramos and Fontana 1996), SO_2_ steam explosion (Sipos et al. 2009), acid treatment (Sun and Cheng 2002; Gamez et al. 2006) and alkali treatment (Chang and Holtzapple 2000; Kaar and Holtzapple 2000).

#### Biological treatment

The brown rot, white rot and soft-rot fungi such as *Phanerochaete chrysosporium*, *Trametes versicolor*, *Ceriporiopsis subvermispora*, *and Pleurotus ostreatus* are employed for biological pretreatment of lignocellulosic biomass. Besides lignin peroxidases and manganese-dependent peroxidases, polyphenol oxidases, laccases and quinosine-reducing enzymes also degrade lignin by producing aromatic radicals. Biological treatment requires low energy and normal environmental conditions but the hydrolysis yield is low and requires long treatment times (Brown 2003).

### Enzymatic saccharification of pretreated biomass

Cellulose hydrolysis, also known as saccharification, is the process in which the cellulose is converted into glucose. Enzymatic hydrolysis is the key to cost-effective ethanol production from lignocellulosic substrates in the long run, as it is very mild process, gives potentially high yields, and the maintenance costs are low compared to acid or alkaline hydrolysis (Kuhad et al. 1997). The process is compatible with many pretreatment methods, but materials poisonous to the enzymes need to be removed or detoxified when chemical pretreatment precedes enzymatic hydrolysis. Factors affecting enzymatic saccharification process involve substrate concentration, enzyme loading, temperature and time of saccharification (Tucker et al. 2003).

#### Cellulase enzyme complex

**Fig. 1:**
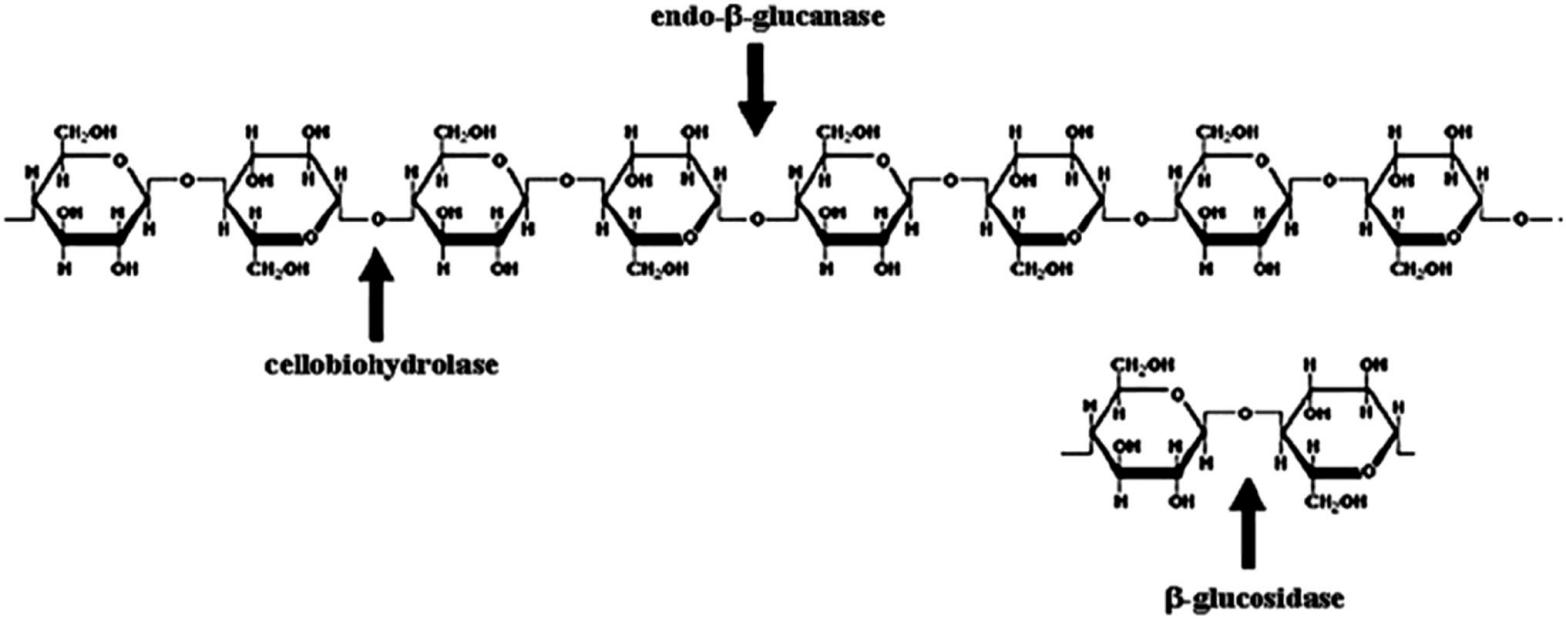
Sites of action of cellulases on cellulose polymer

The cellulose-degrading enzymes were discovered by Reese (1976). The term cellulase complex normally refers to a set of enzymes involved in complete cellulose hydrolysis. Cellulose and the modified cellulose-degrading enzymes are divided into three major groups of enzymes: endo-glucanases (EG), exoglucanases (cellobiohydrolases, CBH) and β-glucosidase (BGL) (Fig. 1) which belong to the EC 3.2.1.X class.

##### Endoglucanases

Endo-β-(1,4)-glucanases (or 1,4-β-d-glucan-4-glucanohydrolases, EC 3.2.1.4), commonly referred to as endoglucanases, are characterized by their random hydrolysis of β-(1,4)-glucosidic linkages (Wood and McCrae 1979). Acting on soluble cellulose derivatives, their random cleavage causes rapid decrease in chain length and hence changes in viscosity relative to the release of reducing end groups. When acting on cellodextrins, the rate of hydrolysis increases with the degree of polymerization within the limits of substrate solubility, with cellobiose and cellotriose being the major final product.

##### Exoglucanases

Exo-β-(1-4)-glucanase (or 1,4-β-d-glucan cellobiohydrolases, EC 3.2.1.91) cleave cellobiose units from the non-reducing ends of cellulose molecules. Exo-β-(1,4)-glucosidase (or 1,4-β-d-Glucan glucohydrolases, EC 3.2.1.74) cleaves glucose units successively from the non-reducing end of the glucan. They are distinguished from β-glucosidase by their preference for substrates of longer chain length and by the inversion of their products.

##### β-glucosidases

β-glucosidase (or β-d-glucoside glucohydrolase, EC 3.2.1.21) hydrolyzes cellobiose and other very short chain β-1,4-oligoglucosides up to cellohexaose to form glucose. Most β-glucosidases are active on a range of β-dimers of glucose. Unlike exoglucosidases, the rate of hydrolysis of cellobiose decreases markedly as the degree of polymerization of the substrate increases.

#### Hemicellulolytic enzymes

In xylan degradation, endo-1,4-β-xylanase, β-xylosidase, α-glucuronidase, α-l-arabinofuranosidase and acetylxylan esterase act on different heteropolymers, while during glucomannan degradation, β-mannanase and β-mannosidase cleave the polymer backbone (Niehaus et al. 1999).

#### Synergism between cellulases

**Fig. 2:**
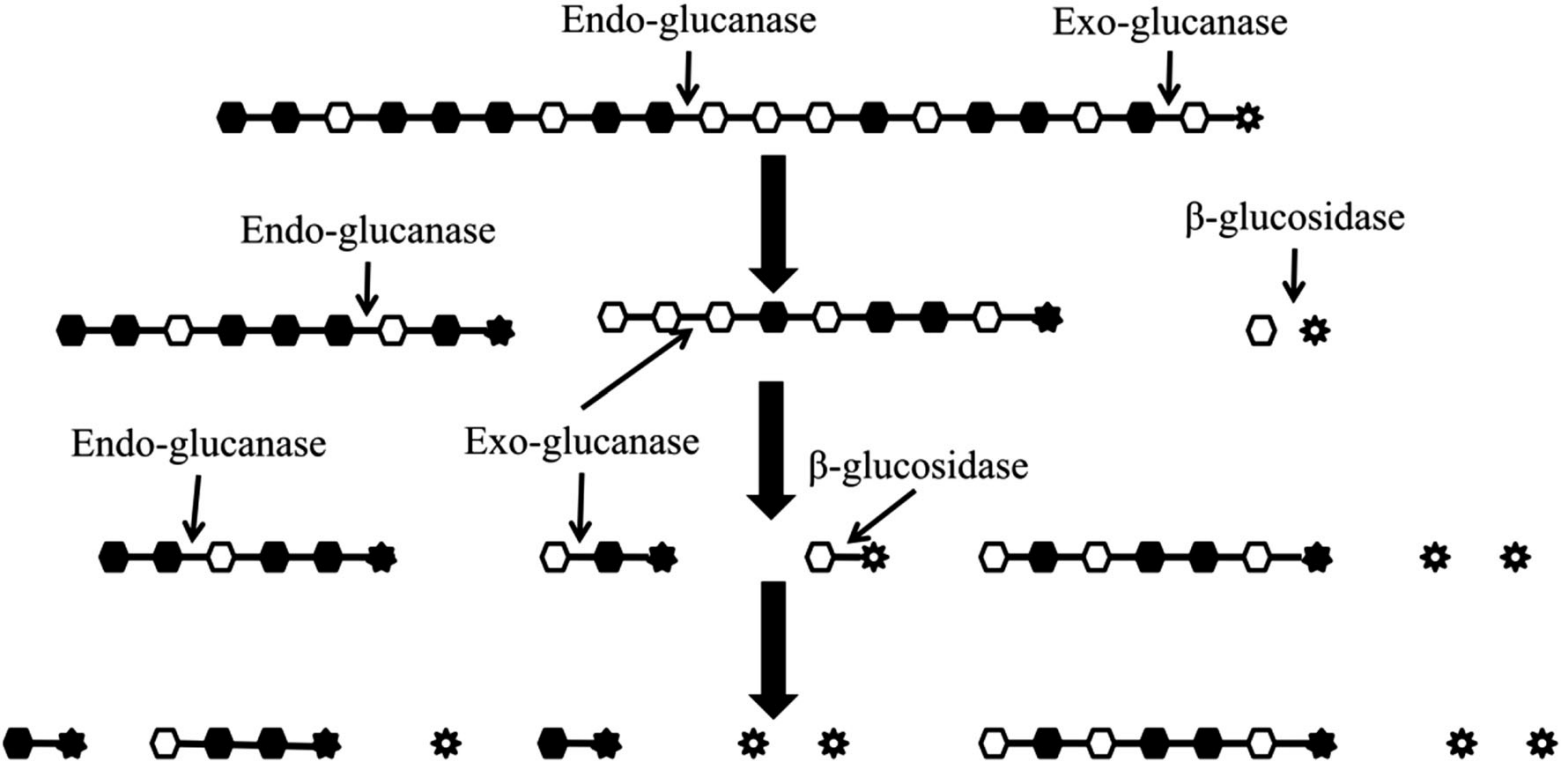
The endo-exo model for synergy between endoglucanase, exoglucanase and β-glucosidase in a cellulolytic system during cellulose hydrolysis.  = reducing end;  = modified reducing end;  = β(1,4) linkage;  = modified glucose;  = unmodified glucose

When the combination of two enzymes is more efficient than the sum of the enzymes acting alone, the two enzymes have synergy. The complete hydrolysis of cellulose to glucose requires a combination of enzymes (endo-, exo-glucanse and β-glucosidase) which work in a synergistic manner for hydrolysis of both native and modified cellulose (Irwin et al. 1993). Presumably the EG makes internal cuts in the cellulose chain, and thereby provides new accessible chain ends for the cellobiohydrolase/exoglucanase and β-glucosidase to work on to gain increased hydrolytic activity (Fig. 2) and due to synergistic effect, each enzyme speeds up the action of the other, with a resulting increase of hydrolysis yield. This model for the synergy between endoglucanases and exoglucanases is called the endo-exo model (Beguin and Aubert 1994).

#### Microbial cellulases

In nature there are many microorganisms that produce cellulolytic enzymes (cellulases). The cellulolytic organisms can be sorted into two different subcategories depending on their enzyme organization in the cell: (a) the microorganism with their cellulases organized into multi-enzyme complexes called cellulosomes, e.g. *Clostridium thermocellum* and *Cellulomonas.* (b) The cellulolytic organisms producing non-complexed cellulase that are not attached to one another, and act individually and cooperatively on cellulose, and by doing this gain strong synergy effects. Examples of fungi from this class are *T. reesei* and *Humicola grisea* and of bacteria, *Streptomyces lividans* and *Cellulomonas fimi* (Bayer et al. 1998). *Trichoderma* spp. (e.g. *T. reesei*, *T. viride*, *T. longibrachiatum*, *T. pseudokoningii* and *T. harzianum*) are ideal cellulolytic model organisms for studying cellulose degradation since these secrete large amounts of cellulases. To date, two CBHs (Cel6A and Cel7A), and at least five EGs (Cel5A, Cel7B, Cel12A, Cel45A, and Cel61A), have been found in the cellulolytic system of *T. reesei*. These enzymes belong to six different GH families, 5, 6, 7, 12, 45, and 61 (Table 4). Today several species of cellulase producing *Penicillium* spp. are known (e.g. *P. citrinum P. occiantalis*, *P. italicum*, etc.). Moreover, many species of *Aspergillus* such as *A. nidulans*, *A. niger* and *A. oryzae* are also known as potential cellulase producers (Pocas-Fonseca and Maranhac 2005).

**Table 4:** Properties of *T. reesei* Cellulases

Enzyme	New name	Molecular mass (kDa)	pI	Conc (%)^b^	Stereo-selectivity	No. of residues	Position of CBM
CBH I	Cel 7A	59–68	3.5–4.2	50–60	Retaining	497	C
CBH II	Cel 6A	50–58	5.1–6.3	15–18	Inverting	447	N
EG1	Cel 7B	50–55	4.6	12–15	Retaining	436	C
EG II	Cel 5A	48	5.5	9–11	Retaining	397	N
EG III	Cel 12A	25	7.4	0–3	Retaining	218	Na^b^
EG IV	Cel 45A	37^a^	Na	na	Na	344	C
EG V	Cel 61A	23^a^	2.8–3.0	0–3	Inverting	270	C
BGL I	Cel 3A	71	8.7	Na	Na	Na	Na
BGL II	Cel 1A	114	4.8	Na	Na	Na	Na

*Ref* Tolan (2002)

*CBH* cellobiohydrolase, *EG* endoglucanase, *BGL* beta-glucosidase

^a^calculated according to the amino acid sequence deducted from gene sequence

^b^Cel 12A does not have a Cellulose binding module

#### Optimization of culture conditions for cellulase production

Cellulase production using fungal cultures is a complex system. Many factors affect cellulase production including nutrient availability, pH, temperature, dissolved oxygen concentration, agitation speed, etc.

##### Medium composition

No general medium composition can be given for growth and optimum cellulase production by all microbes, since the medium must be adapted to the organism in use. Basal medium after Mandels and Weber (1969) has been most frequently used for cellulase production by *T. viridae*, either directly or with slight modifications. Among the cellulosic materials, sulfite pulp, printed papers, mixed waste paper, wheat straw, paddy straw, sugarcane bagasse, jute stick, carboxymethylcellulose corncobs, groundnut shells, cotton, ball milled barley straw, delignified ball milled oat spelt xylan, larch wood xylan, etc. have been used as the substrates for cellulase production. Carboxymethycellulose or cereal straw (1 %, w/w) has been reported as the best carbon source for CMCase and β-glucosidase production using *Chaetomium globosum* as the cellulolytic agent. Further, 3 % malt extract or water hyacinth has also been optimum for CMCase, FPase and β-glucosidase as observed with lactose as the additional carbon sources. However, the saccharification of alkali-treated bagasse at higher substrate levels (up to 4 % w/v) has also been reported. Addition of Ammonium sulfate (0.5 g/l) leads to maximum production of cellulases. However, an increase in the level of β-glucosidase but decrease in endoglucanase and exoglucanase levels was reported when corn steep liquor (0.8 % v/v) was added to synthetic cellulose, wheat straw and wheat bran as the substrates. Phosphorus is an essential requirement for fungal growth, metabolism and several intracellular processes. Different phosphate sources such as potassium dihydrogen phosphate, tetra-sodium pyrophosphate, sodium β-glycerophosphate and dipotassium hydrogen phosphate have been evaluated for their effect on cellulase production (Garg and Neelakantan 1982).

##### Temperature

Temperature has a profound effect on lignocellulosic bioconversion. The temperature range for cellulase production is generally within 25–35 °C for a variety of microbial strains, e.g. *T. reesei*, *Thielavia terrestris*, *Mycelieopthora fergussi*, *Aspergillus wentii*, *Penicillum rubrum*, *Aspergillus niger*, *Aspergillus ornatus* and *Neurospora crassa.* Some of the thermophilic fungi, having maximum growth at or above 45–50 °C, had produced cellulase with maximum activity at 50–78 °C (Li et al. 2011).

##### pH of medium

pH has been known to affect enzyme and cells metabolism tremendously. When the environmental pH is over the operational pH (pH 2.0–pH 7.0), the intracellular pH and enzyme activity are greatly influenced. For cellulase production by *T. reesei* using sugarcane bagasse, an optimum pH range of 5.0–6.0 has been suggested. pH cycling method has been used successfully in the past to obtain high cellulase productivity from 25.0 to 38.75 IU/l/h using 3 % cellulose and *T. reesei* QM 9414. In addition, the fungal cellulases have been found to have the highest catalytic capability between pH 3.5 and 6.0 at 50 °C (Bracey 1998; Kansoh 1998).

##### Oxygen concentration

Oxygen is required for cell growth of most eukaryotes; therefore, cell growth is affected by agitation tremendously. For cellulase production, the percentage of dissolved oxygen is typically maintained above 30 %. Cells die when oxygen is not enough and they stop growing afterwards.

#### Cellulolytic fungal consortium

Although significant advances have been made, a considerable amount of work is still required to enhance the production efficiency of cellulase enzymes. One possible way of improving this situation is to have a mixture containing enzymes of different origins (fungal and/or bacterial). Improved enzyme production by coculturing of two or more microbial strain is being used increasingly for enhanced enzyme production (Garcia-Kirchner et al. 2005). Enhanced cellulose hydrolytic activities have also been observed by the co-cultivation of *A. ellipticus* and *A. fumigatus* (Gupte and Madamwar 1997), co-culture of *A. flavus* and *A. niger* (Saini et al. 2013), co-culture of *A. niger* and *T. reesei* and co-culture of *T. reesei* and *A. phoenicis* (Gutierrez-Correa and Tengerdy 1997).

#### Purification and characterization of fungal cellulases

Culture filtrates from fungal growth often contain a mixture of several extracellular enzymes besides cellulases and hemicellulases and present considerable purification problems. Therefore, multiple purification steps, including different chromatographic runs, are needed to purify cellulase components (Stahlberg et al. 1988). Various chromatographic techniques have been described in the literature for purification of cellulase enzyme such as molecular exclusion, affinity chromatography, ion-exchange chromatography, chromatofocusing, fast protein liquid chromatography (FPLC) and hydrophobic interaction chromatography (HIC) (Tomaz and Queiroz 1999). The biochemical characteristics of β-glucosidase and cellulase are summarized in Table 5.

**Table 5:** Biochemical properties of fungal β-glucosidase and cellulases

Source	Mr (kDa)	Quaternary structure	Opt. pH	Opt. Temp. (°C)
β-glucosidases				
*Aspergillus niger*	105	Dimer	5	55
*Aspergillus niger*	330	Tetramer	4.6–5.3	70
*Candida peltate*	43	Monomer	5	50
*Ceriporiopsis subvermispora*	110	NR	3.5	60
*Fomitipsis pinicola*	105	Monomer	4.5	50
*Melanocarpus *sp.	92	Monomer	6	60
*Phanerochaete chrysosporium*	114	NR	4–5.2	NR
*Penicillium purpurogenum*	110	Monomer	5	65
Cellulases				
*T. reesei*	48	Monomeric	4.0–5.0	50
*A. niger*	31	Monomeric	4.0	30
*A. nidulans*	83	NR	5.0	50
*T. harzianum*	78	Monomeric	7.7–8.0	40
*Geotrichum* sp.	80	Monomeric	5.5	55
*Trichoderma* sp.	51	Monomeric	5	50
*F. oxysporum*	42.7	Monomeric	5.0	75

*Ref* Rashid and Siddiqui (1998)

*NR* not reported

#### Commercial cellulases

The cost of cellulase enzymes is widely considered an important factor in the commercialization of lignocellulosic biomass-to-ethanol processes (Wright 1988). A large number of industries are manufacturing cellulase enzymes globally (Table 6). The enzyme producers Genencor International and Novozymes A/S have achieved increased enzyme activity and reduced 30-fold cost of production. Genencor developed a blend of genetically enhanced enzymes that act in synergy to convert cellulose to sugars (http://www.nrel.gov/awards/2004hrvtd.html). Cellic CTec3 is a state-of-the-art cellulase and hemicellulase complex that allows for the most cost-efficient conversion of pretreated lignocellulosic materials to fermentable sugars compared to any other cellulase or enzyme complex available in the market for cellulosic ethanol production (http://www.bioenergy.novozymes.com/en/cellulosic-ethanol/CellicCTec3/Pages/default.aspx).

**Table 6:** Commercially available cellulases

Product name	Company	Source	pH	Temp (°C)	Form
Biocellulase TRI	Quest Intl. (USA)	*T. reesei*	4.0–5.0	50	Liquid
Biocellulase A	Quest Intl. (USA)	*A. niger*	5.0	55	Powder
Celluclast 1.5L	Novo Nordisk, (Danbury, CT)	*T. reesei*	5.0	50	Liquid
Cellulase TAP10^6^	Amano Enzyme (Troy, VA)	*T. viride*	5.0	50	Powder
Cellulase AP30 K	Amano Enzyme (Troy, VA)	*A. niger*	4.5	60	Powder
Cellulase TRL	Solvay Enzymes (Elkhart, IN)	*T. reesei*	4.5	50	Liquid
Econase CE	Alko-EDC (USA)	*T. reesei*	5.0	50	Liquid
Multifect CL	Genencor Intl. (USA)	*T. reesei*	4.5	50	Liquid
Multifect GC	Genencor Intl. (USA)	*T. reesei*	4.0	50	Liquid
Spezyme	Genencor Intl. (USA)	*T. reesei*	4.0	50	Liquid
Ultra-Low Microbial (ULM)	Iogen, (Ottawa, Canada)	*T. reesei*	NA	NA	Liquid
Cellic CTec 2	Novozymes (Bagsvaerd, Denmark)	Enzyme cocktail	NA	NA	Liquid
Cellic CTec 3	Novozymes (Bagsvaerd, Denmark)	Enzyme cocktail	NA	NA	Liquid

*Ref* Nieves et al. (1998)

#### Visualizing structural changes in hydrolyzed bagasse

To detect morphological and structural changes in polymers, some physico-chemical (thermal analysis, X-ray diffraction, gel permeation chromatography), spectroscopic (Infrared and Raman spectroscopy, nuclear magnetic resonance and mass spectroscopy) and microscopic [scanning electron microscopy (SEM), atomic force microscopy (AFM), transmission electron microscopy (TEM), and chemical force microscopy (CFM)] (Samir et al. 2005) methods are consistently being used in the literature (Volke-Spulveda 1998). Standard methods generally employed to examine biodegradation of biopolymers are: visual observations, weight loss measurements through determination of residual polymer, changes in mechanical properties and molar mass and radio-labeling. A number of other techniques have also been used to assess the biodegradability of polymeric material. These include differential scanning colorimetry (DSC), nuclear magnetic resonance spectroscopy (NMR), X-ray photoelectron spectroscopy (XPS), X-ray Diffraction (XRD), contact angle measurements and water uptake Carmen et al. (2009). More recently, Fourier transform infrared spectroscopy (FTIR) and simultaneous TG-DTG-DTA have been used to study biodegradation of polymers (Hadad et al. 2005).

### Fermentation and product recovery

The biomass is hydrolyzed by cellulolytic enzymes into fermentable sugars (pentoses or hexoses), which are fermented to ethanol by several microorganisms. For making ethanol production commercially viable, an ideal microorganism should utilize broad range of substrates, with high ethanol yield, titre and productivity, and should have high tolerance to ethanol, temperature and inhibitors present in hydrolysate. Some of the major characters of a viable ethanol process are listed in Table 7.

**Table 7:** Important traits for bioethanol fermentation process

Trait	Requirement
Bioethanol yield	>90 % of theoritical maximum
Bioethanol tolerance	>40 g/l
*Y*_P/S_	Close to 0.5 g/g
*Q*_P_	>1 g/l/h
Robust growth and simple growth requirement	Inexpensive medium formulation
Culture growth conditions retard contaminants	Acidic pH or higher temperatures

*Ref* Dien et al. (2003), Balat et al. (2008)

### Integrated bioprocesses for saccharification and fermentation

**Fig. 3:**
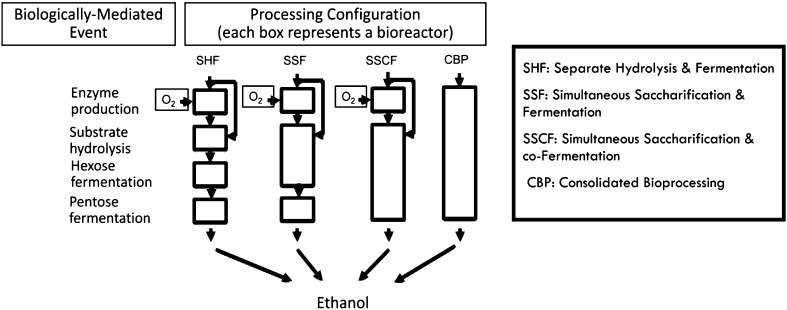
Process configurations for conversion of lignocellulosic biomass to bioethanol

As shown in Fig. 3, various saccharification and fermentation bioprocess integrations have been reported. First is separate (or sequential) hydrolysis and fermentation (SHF), a two-stage process involving saccharification of the substrate, followed by the fermentation of saccharified fluid, separately. Main features of SHF include optimal operating conditions for each step and minimal interactions between hydrolysis and fermentation steps. However, SHF process is limited by end-product inhibition and chances of contaminations, which may decreases ethanol yield (Balat et al. 2008; Sanchez and Cardona 2008; Neves et al. 2007; Sarkar et al. 2012). Second process configuration is simultaneous saccharification and fermentation (SSF), in which hydrolyzes of cellulose is consolidated with the direct fermentation of the produced glucose, avoiding the problem of product inhibition associated with enzymes. Main advantages of simultaneous saccharification and fermentation process are comparatively lower costs, higher ethanol yields due to removal of feedback inhibition on enzymatic saccharification and reduction in the required number of vessels or reactors. Some of the disadvantages of SSF are different optimum conditions for enzyme hydrolysis and fermentation processes (Bjerre et al. 1996; Hamelinck et al. 2005; Neves et al. 2007; Balat et al. 2008; Sarkar et al. 2012). The most common and robust fermenting microorganisms employed in ethanol production are *S. cerevisiae* and *Z. mobilis*. Ethanol production from sugars derived from starch and sucrose has been commercially dominated by this yeast. However, *S. cereviseae* is capable of converting only hexose sugars to ethanol. The most promising yeasts that have the ability to use both pentoses and hexoses are *Pichia stipitis*, *Candida shehatae* and *Pachysolan tannophilus*. Thermotolerant yeasts, such as *Kluyveromyces marixianus*, could be more suitable for ethanol production at industrial level, because of their ability to ferment at higher temperatures. In high-temperature process energy savings can be achieved through a reduction in cooling costs. Hence, thermotolerant yeasts are highly desirable in SSF process. Another strategy for ethanol fermentation is simultaneous saccharification and co-fermentation (SSCF), in which co-fermentation of hexoses and pentoses is carried out. In SSCF the co-fermenting microorganisms need to be compatible in terms of operating pH and temperature (Neves et al. 2007). However, the ability to ferment pentoses along with hexoses is not widespread among microorganisms and lack of ideal co-fermenting microorganism is one of the greatest obstacles in industrial production of second-generation ethanol (Talebnia et al. 2010). Sometimes co-culture technique proves to be a useful technology whereby a combination of hexose and pentose fermenting microorganisms is utilized for complete utilization of biomass sugars. For example, co-culture of *Candida shehatae* and *Saccharomyces cerevisiae* was reported as suitable for the SSCF process (Neves et al. 2007). One more configuration for ethanol fermentation is consolidated bioprocessing (CBP). In this process, ethanol and all required enzymes are produced by a single microorganism or microbial community, in the same reactor. The process is also known as direct microbial conversion (DMC). The main advantage of CBP is that its application avoids the cost involved in purchase or production of enzymes (Hamelinck et al. 2005; Lynd et al. 2005). Approached pathways in the development of CBP organisms are described by Lynd et al. (2002). Bacteria such as *Clostridium thermocellum* and some fungi including *Neurospora crassa*, *Fusarium oxysporum* and *Paecilomyces* sp. have shown this type of activity. However, CBP is a less-efficient process with poor ethanol yields and longer fermentation time of more than 3–4 days. Significant cost reductions are expected when progressing from improved SSF via SSCF to CBP. Table 8 summarizes the bioethanol production from some major agroresidues.

**Table 8:** Bioethanol production from major agroresidues

Biomass	Fermenting microorganism	Ethanol yield or titre
Wheat straw	*Pichia stipitis* NRRL Y-7124 (strain adapted to acid hydrolysate inhibitors)	0.35 g/g yield
	*Pichia stipitis* A	0.41 g/g yield
Rice straw	*Candida shehatae* NCL-3501 (co-ferment glucose and xylose)	0.45 g/g and 0.37 g/g from autohydrolysate and acid hydrolysate, respectively
	*S. cerevisiae* ATCC 26603 (only ferment glucose)	
SCB	*Pichia stipitis* BCC15191 (glucose–xylose co-fermenting strain)	0.29 g/g yield
	Genetically modified *E. coli* KO11 (glucose and xylose co-fermenting strain)	91.50 % yield and 3.15 % (w/v) ethanol titre

*Ref* Sarkar et al. (2012)

### Optimization of saccharification and fermentation bioprocess

Experimental design and statistical analysis for optimization of process conditions are some of the most critical stages in the development of an efficient and economic bioprocess. Classical (such as one factor at a time) and statistical methodologies are available for optimizing process conditions (such as response surface methodology, RSM). RSM is an efficient statistical technique for optimization of multiple variables to predict the best performance conditions with a minimum number of experiments. These designs are used to find improved or optimal process settings, troubleshoot process problems and weak points and make a product or process more robust against external and noncontrollable influences. Full factorial, partial factorial, Box-Behnken and central composite rotatable designs (CCRD) are the most common techniques used for process analysis and monitoring (Sasikumar and Viruthagiri 2008). This method has been in use for hydrolysis of a wide variety of materials to find the optimum conditions for different lignocellulosic biomasses (Talebnia et al. 2010; Saini et al. 2013) and for standardizing SSF for production of ethanol from pretreated sugarcane bagasse by cellulase and yeast *Kluyveromyces fragilis* (Sasikumar and Viruthagiri 2008).

## Economic considerations for cellulosic ethanol production

To be competitive, and economically acceptable, the cost for bioconversion of biomass to liquid fuel must be lower than the current gasoline prices (Subramanian et al. 2005). It seems, however, much more attainable because of increasing efforts of researchers working towards improvisation in the efficiency of biomass conversion technologies. However, there is still huge scope to bring down the cost of biomass-to-ethanol conversion. The cost of feedstock and cellulolytic enzymes are the two important parameters for low-cost ethanol production. Biomass feedstock cost represents around 40 % of the ethanol production cost. An analysis of the potential of bioethanol in short and long term (2030) in terms of performance, key technologies and economic aspects such as cost per kilometer driven has been conducted recently by Hamelinck et al. (2005).

The choice of feedstock for ethanol production depends upon its availability and the ongoing uses. Some dedicated energy sources like damaged rice, sorghum grains and sweet sorghum bagasse, sunflower stalks and hulls, *Eicchornia crassipies*, *P. brava,* alfalfa fibers, residual starch and crushed wheat grains, agro waste, and *Saccharum spontaneum* are more feasible sources for bioethanol production. The use of integrated approach (Process engineering, fermentation and enzyme and metabolic engineering) could improve the ethanol production economics. Aristidou and Penttila (2000) reported that the total cost of cellulosic ethanol will be dropped from more than $1.0 to ~$0.3–0.5/l, with a projected cost of less than $0.25/l in the near future. Wooley et al. (1999) have explained the further economic analysis of bioethanol ($ 0.78/gallon) and suggested a projected cost of as low as $ 0.20/l by 2015 if enzymatic processing and biomass improvement targets are met. The projected cost of ethanol production from cellulosic biomass as per the earlier estimates ($4.63/gallon in 1980) has been reduced by almost a factor of four ($1.22/gallon) over the last 20 years.

The distillation cost of per unit amount of ethanol produced is substantially higher at low ethanol concentrations; the researchers have dealt with the idea of concentrating sugar solutions prior to fermentation. Ethanol distillation cost can be further improved using membrane distillation process. It has the lowest operational cost, simple to use and is easy to maintain and is the most efficient and cost-effective option among the available distillation processes (Camacho et al. 2013).

## Challenges and future outlook

Lignocellulosic biomass has long been advocated as a key feedstock for cost-effective bioethanol production in an environment-friendly and sustainable manner. Lignocellulose-rich agricultural wastes/residues are abundant and renewable resources for second-generation bioethanol production. Till now research on utilization of agricultural residues for second-generation bioethanol production has shown very promising results worldwide. Several lab and pilot scale as well as demonstration studies for cellulosic ethanol production from agrowastes have been reported successful but still there exists a huge gap between the projected and actual bioethanol production at industrial level. Therefore, to make full use of these cheap, abundant and renewable resources for economically feasible bioethanol production, several difficulties have yet to be overcome. These challenges include (1) collection, harvesting, supply and handling of agrowastes; (2) cost-effective pretreatment technology; (3) reduction in cost of cellulolytic enzymes; (4) achieving efficient depolymerization of cellulose and hemicellulose into fermentable monomeric sugars by development of more efficient enzyme blends/cocktails; (5) use of higher biomass loadings for achieving higher yields of fermentable sugars and thus high titers of ethanol; (6) use of efficient thermotolerant yeast strains capable of fermentation at temperatures more close to optimum for cellulolytic enzymes; and finally, (7) xylose and glucose co-fermentation, and the use of recombinant/metabolically engineered microbial strains. Considering the huge availability of feedstocks from agriculture and other sources and tremendous efforts being carried out to make second-generation biofuel production more cost-effective, there seems huge scope for the large-scale production of second-generation biofuels in near future. This will certainly involve elimination of the current technology hurdles of lignocellulose to bioethanol conversion process by making microbial processes more efficient.

## Conclusions

Lignocellulosic biomass-derived second-generation biofuels are promising alternatives to petroleum-based fossil fuels. The utilization of agricultural residues and wastes for bioethanol production is a cost-effective and environmental-friendly approach for sustainable development. Considering the recent research progress in the fields of enzyme production, pretreatment, as well as metabolic engineering of yeasts, production of bioethanol from lignocellulosic agricultural wastes will certainly prove to be a feasible technology to achieve energy security in very near future.
